# Evaluation of Chosen Cytokine Levels among Patients with Herpes Zoster as Ability to Provide Immune Response

**DOI:** 10.1371/journal.pone.0150301

**Published:** 2016-03-02

**Authors:** Agata Zajkowska, Adam Garkowski, Renata Świerzbińska, Alina Kułakowska, Monika Emilia Król, Iwona Ptaszyńska-Sarosiek, Anna Nowicka-Ciełuszecka, Sławomir Pancewicz, Piotr Czupryna, Anna Moniuszko, Joanna Zajkowska

**Affiliations:** 1 Department of Neurology, Medical University of Białystok, Białystok, Poland; 2 Department of Radiology, Medical University of Białystok, Białystok, Poland; 3 Department of Infectious Diseases and Neuroinfections, Medical University of Bialystok, Białystok, Poland; 4 Department of Forensic Medicine, Medical University of Białystok, Białystok, Poland; 5 Department of Infectious Diseases, District Hospital in Hajnówka, Hajnówka, Poland; UC Irvine Medical Center, UNITED STATES

## Abstract

**Aim and Background:**

Herpes zoster is a viral disease caused by the reactivation of varicella–zoster virus (VZV) which remained latent in the cranial nerve or dorsal root ganglia. Cell-mediated immunity is known to decline with age as part of immunosenescence and can lead to the reactivation of VZV. Whereas herpes zoster is usually mild in healthy young persons, older patients are at increased risk for complications. In the present study we investigated the serum cytokine profile (IL-17, IL-23, IL-21, IL-4, IL-12), representing cellular and humoral immunity and assessed the level of VZV IgG antibodies in patients with herpes zoster.

**Methods:**

We investigated the serum concentrations of IL-17, IL-23, IL-21, IL-4, IL-12 and the level of VZV IgG antibodies in 23 patients with herpes zoster who did not develop superinfection. The control group was represented by 21 individuals in similar age with no inflammatory and infectious diseases. Cytokine and antibodies levels were measured by ELISA method. Statistical analysis was performed using the ROC curve (receiver operating characteristic), t-test, Welch’s *t*-test, and nonparametric tests with STATISTICA 10 software.

**Results:**

In patients with herpes zoster, the serum level of IL-17, IL-23, IL-21, IL-4 and IL-12 as well as VZV IgG antibodies titer were statistically significantly increased compared to control group.

**Conclusion:**

Our results confirm the broad activation of the immune system involving humoral and cell-mediated immunity.

## Introduction

Varicella zoster virus (VZV) is a neuropathic human alphaherpesvirus that causes two clinically different diseases—chickenpox during primary infection and herpes zoster (HZ), also known as shingles. The virus becomes latent in cranial nerve and dorsal root ganglia after chickenpox and can reactivate many years later in immunosuppressed patients, usually resulting in HZ. The clinical manifestation of HZ is characterized by prodromal pain followed by a vesicular rash in different stages of development that is usually limited to a single dermatome [1]. The disease affects far more frequently older people, especially ones suffering from chronic diseases [2]. Potentially, this particular group of patients may be prophylactically vaccinated against VZV to prevent not only the VZV reactivation, but also the potential severe course of the disease that can occur with e.g. life threatening bacterial superinfection, blindness, central nervous system vasculitis or result in difficult to treat postherpetic neuralgia (PHN) [3]. However, there is an essential condition for the VZV vaccine efficacy and effectiveness: efficient immune system. VZV-specific cell-mediated immunity plays the key role in controlling the latency and reducing the reactivation of the virus [2, 4]. The depressed cell-mediated immunity is well-documented in the elderly and in patients with malignancies. Consequently, the rate of HZ is higher in these groups.

The aims of the current study were the evaluation of a variety of serum cytokines with determination of levels of VZV IgG antibodies in patients with HZ in comparison with the control group.

## Materials and Methods

The study group included 23 patients clinically diagnosed as HZ hospitalized in the Department of Infectious Diseases and Neuroinfections, Medical University of Białystok, Poland. We assessed the serum level of IL-17, IL-23, IL-21, IL-4, IL-12 and the level of VZV IgG antibodies in 23 patients from this group; these patients did not develop secondary bacterial skin infection and had no other inflammatory conditions that could influence the results. The control group consisted of 21 patients in similar age without any inflammatory or infectious diseases, diagnosed in the Department of Neurology, Medical University of Bialystok, Poland. None of the patients received steroids or immunosuppressive treatment before and during the study. The written informed consent was obtained from all study participants including controls.

For serum cytokines and VZV IgG antibodies assays, venous blood samples were obtained from a peripheral vein. The blood samples were collected from the patients during the first day of hospitalization at the beginning of the acute phase of the HZ. All samples were transported to the laboratory immediately after collection. For evaluation of serum level of cytokines, ELISA tests were used: Human IL-4 test Kit Manual (XpressBio, San Diego, USA), Human IL-17 ELISA Kit Manual (XpressBio, San Diego, USA), Human IL-23 ELISA Kit (GenWay Biotech Inc), Human Il-21 ELISA (Kit GenWay Biotech Inc), IL-12 (BD Biosciences USA). To determine the VZV IgG antibodies level against VZV, the Varicella Zoster—Virus IgG ELISA (DRG Diagnostics Germany) test was used. The study was previously approved by the Bioethical Committee of the Medical University of Białystok, Poland.

Statistic analysis was performed with ROC (receiving operating curves), t-test, Welch’s *t*-test, and nonparametric tests with STATISTICA 10 software.

## Results

### Clinical characteristics of the study populations

The patients’ age ranged from 33 to 90 years (the average age of men and women was 68.6 and 64.3 years respectively). Patients who were over 50 years old represented 85% of all patients. There was no statistically significant difference in the average age between the HZ patients and the control group. All patients were Caucasian. In the vast majority of patients, the occurrence of immune-declining conditions were already detected during the initial clinical examination e.g. old age, type II diabetes, malignancies, chronic kidney disease, mechanical trauma or severe psychological stress within the previous 6 months. The skin lesions appeared most commonly in dermatomes innervated by sensory branches from C2 to Th12 (53%). HZ ophthalmicus accounted for 20% of cases; in 17.8% of cases, HZ involved the area of the facial’s nerve innervations; 8.9% of patients presented with HZ oticus with facial nerve palsy. The patient characteristics is presented in Table 1.

**Table 1:** The patients’ characteristic.

Characteristic	Herpes zoster (n = 23)	Control group (n = 21)
Mean age in years (range)	66.5 (33–90)	63.8 (36–84)
Female:male	13:10	10:11
**HZ localization**		
C2-Th12	53%	-
Ophthalmic nerve dermatomes	20%	-
Facial nerve dermatomes	17.8%	-
Ramsay Hunt syndrome	8.9%	-

### Median and range IL-4, IL-12, IL-17, IL-23, IL-21 cytokine concentrations (pg/ml) in herpes zoster (1) and control group (2) and analysis variables using ROC curves

**Fig 1:**
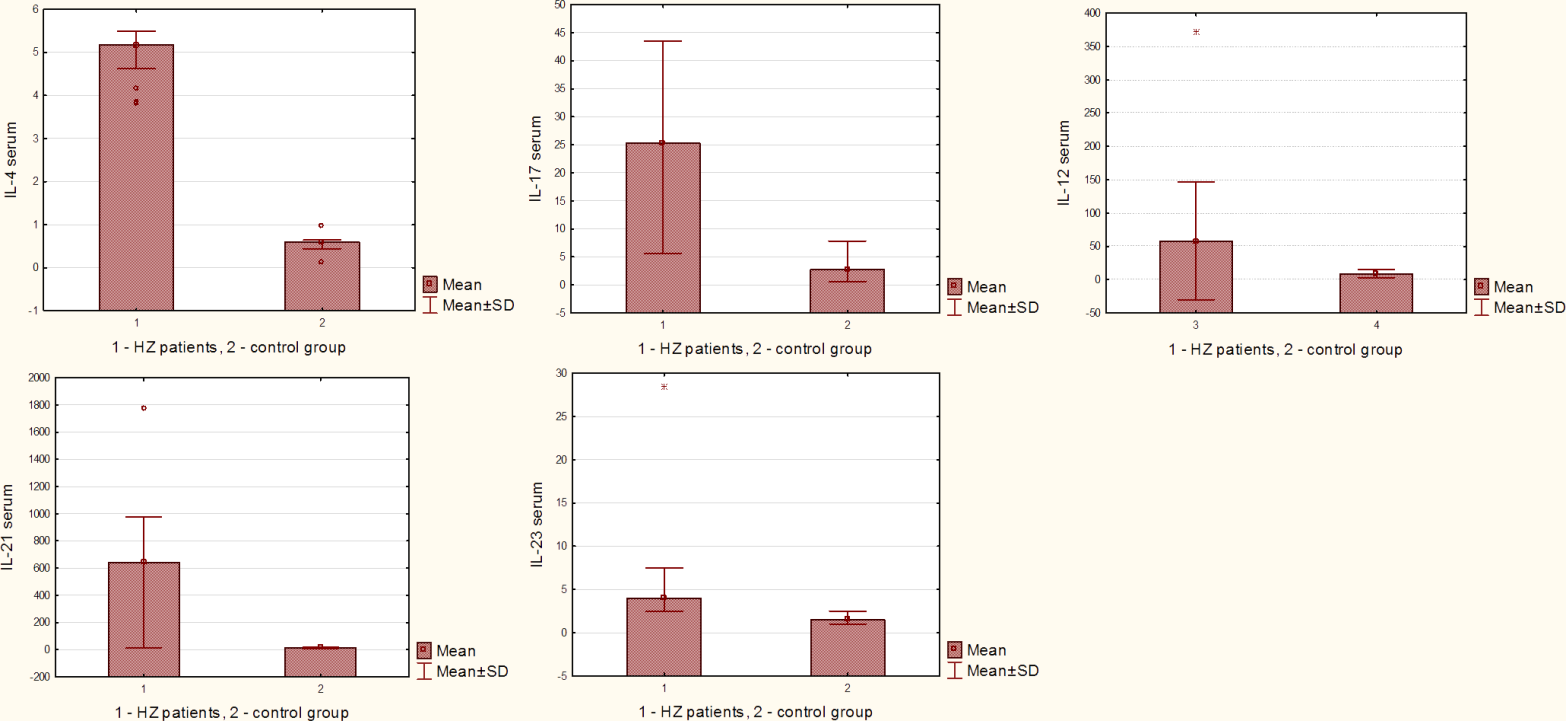
The mean results of IL-17, IL-23, IL-21, IL-4, IL-12 cytokine concentration (pg/ml) in group 1 n = 23 (patients with HZ) and group 2 n = 21 (control group), including x ±SD.

**Fig 2:**
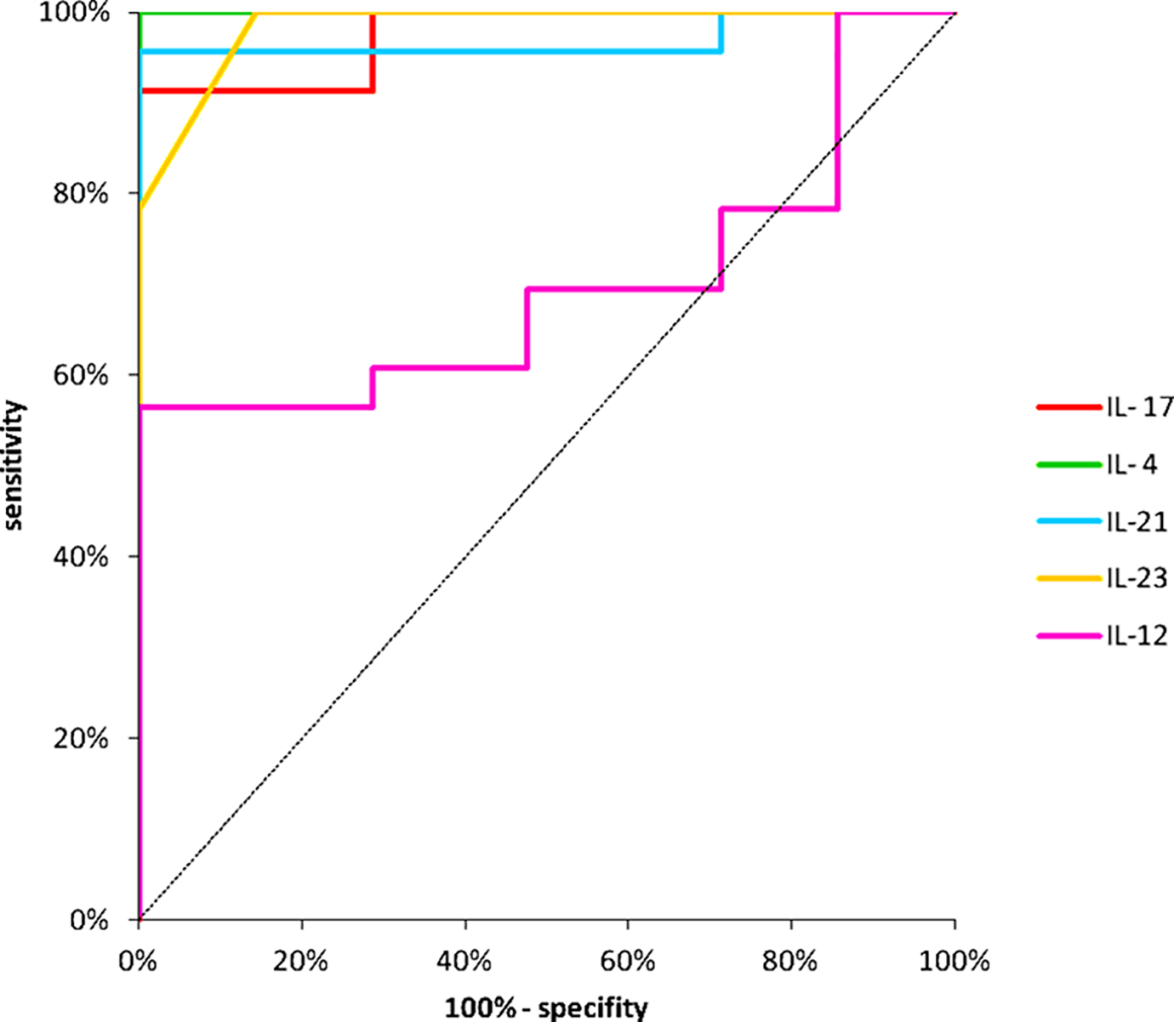
Statistical analysis using ROC curves. Analysis with the ROC curves showed that serum IL-17, IL-23, IL-21, IL-4 and IL-12 differentiate group 1 and group 2 (p<0.05).

The level of circulating IL-4, IL-12, IL-17, IL-23 and IL-21 was significantly elevated in the HZ patients versus normal controls. Serum level of these cytokines in patients with HZ infection and control group is presented in Fig 1 and Table 2A. The analysis of indicator variables and statistical analysis using ROC curves are shown in Fig 2 and Table 2B. ROC curves analysis of IL-17, IL-23, IL-21, IL-4, IL-12 serum cytokines concentration in both group confirms hypothesis that in HZ group cytokines production is essentially higher than in control group.

**Table 2:** A: The results of measured cytokines concentrations (pg/ml), mean, range and median in the group 1 (HZ) and group 2 (control). B: The analysis of indicator variables in serum in herpes zoster and control group (ROC curves).

**A**					
	**Group**	**x**	**SD**	**Min-max**	**median**
**IL-17**	1	24,00*	12,49	5,67–43,4	25
**IL-17**	2	3,47	2,65	0,57–7,43	2,7
**IL-23**	1	5,47*	5,27	2,5–28,5	4,0
**IL-23**	2	1,71	0,46	1,0–2,5	1,5
**IL-21**	1	631,38*	390,0	13,2–1771,0	642
**IL-21**	2	13,7	4,1	5,75–17,133	15,73
**IL-4**	1	5,01*	0,48	3,83–5,49	5,17
**IL-4**	2	0,55	0,24	0,11–0,97	0,59
**IL-12Il-12**	1	74,153*	108,17	0,92–371,13	35,82
**IL-12**	2	8,888	6,55	0,857–19,44	6,52
**B**					
**Variable (pg/ml)**	**n**	**AUC**	**SE**	**95% C.I.(AUC)**	**p (AUC = 0,5)**
**IL-17.**	44	0,9752	0,0193	(0,937–1,013)	0,0000
**IL-23**	44	0,9845	0,0106	(0,964–1,005)	0,0000
**IL-21**	44	0,9689	0,0314	(0,907–1,030)	0,0000
**IL-4**	44	1,0000	0,0000	(1,000–1,000)	0,0000
**IL-12**	44	0,7184	0,0817	(0,558–0,878)	0,0075

* Statistical significance (t-test, Welch’s *t*-test).

### VZV IgG antibodies titers

**Fig 3:**
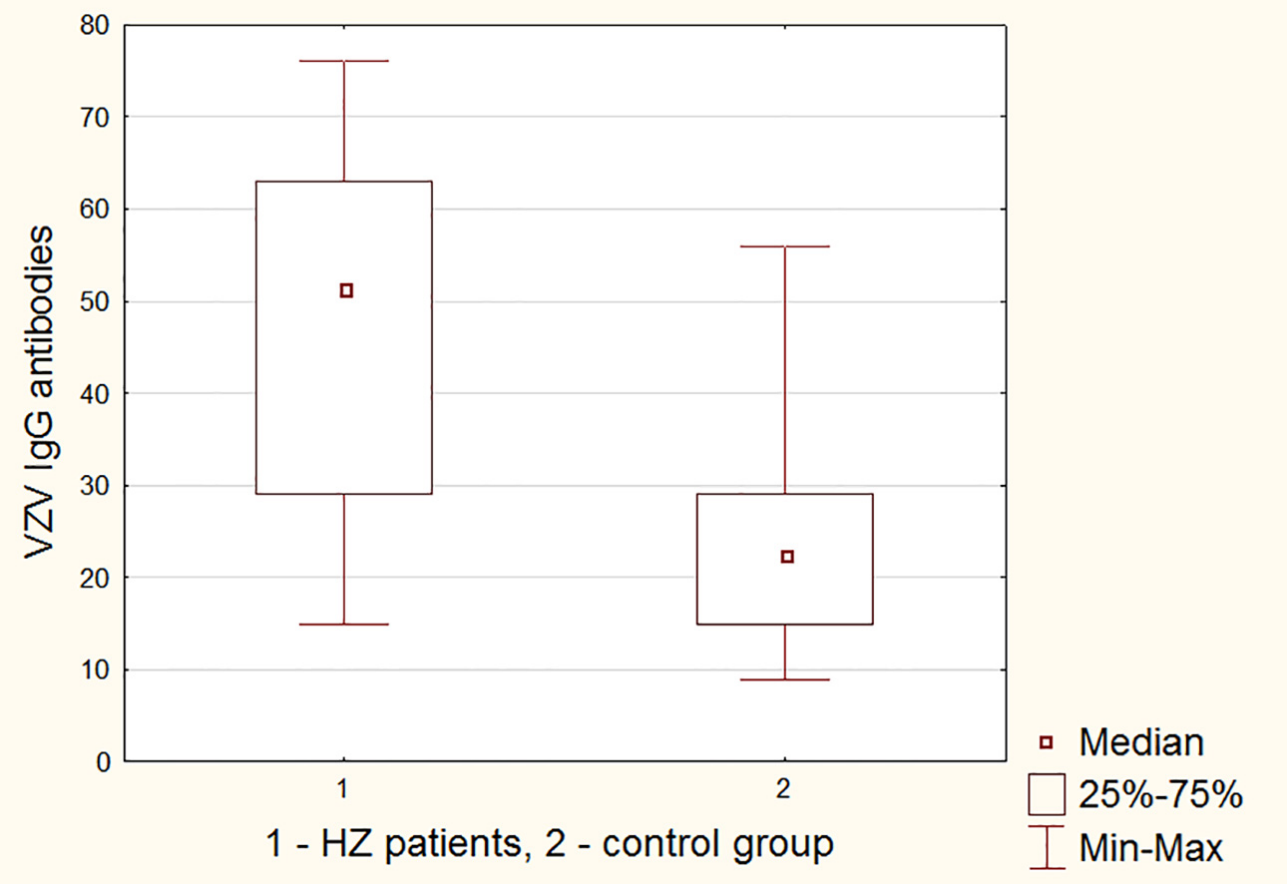
The mean results of VZV IgG antibodies level in group 1 (HZ) and group 2 (control).

The VZV IgG antibody titer of the patients with HZ was elevated significantly (P<0.0001) compared to control group (Fig 3 and Table 3). Thus, as might be expected, the VZV reactivation and the emergence of the HZ lesions boosted the antibody level to the virus.

**Table 3:** The results of measured antibody titers, mean, range and median in the group 1 (HZ) and group 2 (control).

group	x	SD	Min-max	median	P<0,05
**1**	46,39	19,97	15–76	51	p<0,0001
**2**	23,86	12,2	9–56	22,5	p<0,0001

### Correlation of measured cytokines and VZV IgG antibodies level

**Fig 4:**
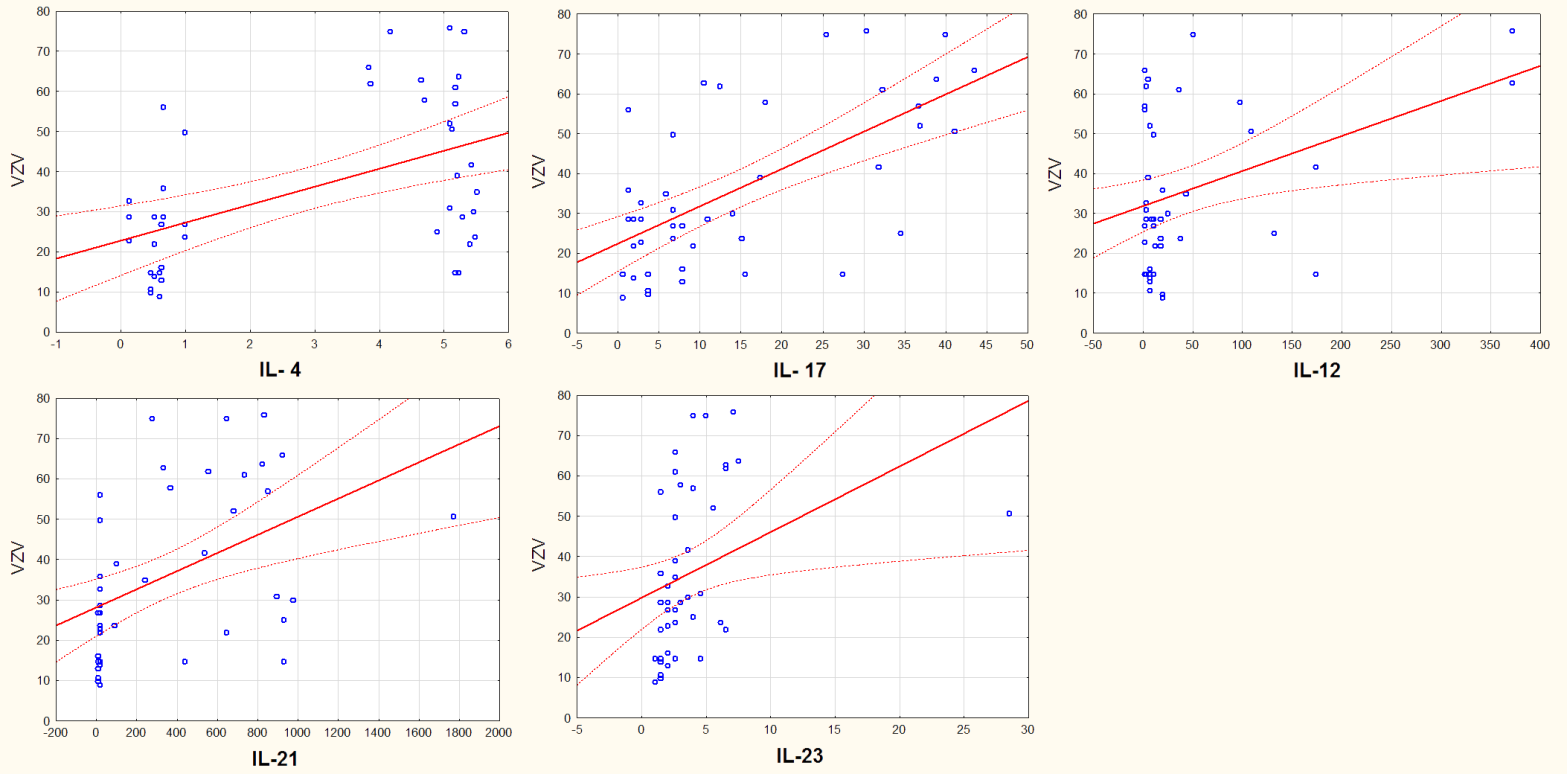
Correlation among cytokine concentration and VZV antibodies.

We observed strong correlation among all serum cytokines and VZV IgG antibodies. The differences were observed in both groups (HZ patients and control group). Strong correlation of IL-17 is observed in group 1 and weak in group 2, negative correlation of IL-4 is observed in group 1. The results are so interesting that some measurements should be performed in time (Table 4 and Fig 4).

**Table 4:** Correlation of measured cytokines and VZV IgG antibodies level.

Correlation of measured cytokines and VZV IgG antibodies level	Group 1 and 2 n = 45		Group 1 n = 23		Group 2 n = 21	
	r	p	r	p	r	p
**IL- 17**	0,64307	0,00000	0,4792	0,479201	0,00681	0,976616
**IL- 4**	0,50749	0,0004	-0,50731	0,507314	0,21545	0,348281
**IL-21**	0,46788	0,0014	0,09909	0,099095	0,48663	0,025283
**IL-23**	0,34299	0,0227	0,12584	0,125841	0,38108	0,088303
**IL-12**	0,3681	0,0140	0,23292	0,232925	-0,17977	0,435545

## Discussion

Cytokines are important mediators of immune responses that allow for the integration of the cellular behaviour in respect immune responses; also, they play an essential role in the expression of cell-mediated immunity. In this study, the serum level of Th2 and Th17 cytokines (IL-4, IL-12, IL-17, IL-21, IL-23) was assessed in HZ individuals. The pathomechanism governing the transition of VZV from latency to reactivation is not yet clear, but it is believed that T cells play a crucial role in the interplay between the immune system and latent VZV infection. The cell-mediated immune response is the most important immune defense mechanism against VZV infection. Alterations in the T-cell-mediated immune response, are of particular relevance for VZV reactivation. This cellular immunity, as contrasted with humoral immunity, decreases with advancing age, which may account for a propensity to reactivation of VZV. Some cytokines in HZ have been well investigated (e.g. IL-2, IL-6, IL-10, IFN-γ and TNF-α), but the others are an ongoing focus of investigation. In this study we chose less known cytokines in HZ, representing broad spectrum of immunological response (cellular and humoral). To our knowledge, other studies assessing the level of IL-17, IL-21 and IL-23 in patients with HZ have not been reported. Our study showed that the mean serum level of these cytokines was significantly higher in patients with HZ than in those of the controls in the calculations using ROC curves. These results confirm the broad activation of the immune system involving humoral and cell-mediated immunity during HZ. Furthermore, amongst the cytokines we measured, the levels of serum IL-4 and IL-17 were the highest.

IL-4 is produced by Th2 cells, mast cells and basophils. It has a broad biological effects, including the induction of T cells differentiation into Th2 cells and IgE class switching in B cells, and the increase of the MHC class II expression on B cells. IL-4 suppresses the production of Th1 cells, IL-1, IL-6, TNF-α and IFN-γ. The overproduction of IL-4 is associated with inflammatory and autoimmune diseases [5]. Elevated amounts of IL-4 have also been detected in scleroderma, asthma and tuberculosis [6–8]. In the study by *Hayward et al*., the concentration of IL-4 was significantly increased in the supernatant of VZV-stimulated cultures of blood lymphocytes—obtained from healthy individuals who had chickenpox ≥15 years earlier, none of whom had HZ [9]. *Zhang et al*. detected an increased level of IL-4 in blister fluid of patients with HZ [10]. In contrast to our results, in the previous study by *Zak-Prelich et al*., serum IL-4 concentration was below the detectable limit in HZ patients [11].

Another cytokine, IL-12, is a proinflammatory protein produced mostly by activated monocytes, macrophages, neutrophils, and dendritic cells in response to antigenic stimulation. It induces production of IFN-γ by Th1 and NK cells. The reduced production of IL-12 impairs Th1 response and increases susceptibility to infection with intracellular pathogens [5, 12]. Unlike our results, in another study, the serum concentration of IL-12 did not differ significantly between the HZ group and the control group [11]. Also, in the study conducted by *Yu et al*., the production of IL-12 by peripheral blood mononuclear cell induced by VZV in healthy human beings was absolutely minimal [13].

IL-17 induces the production of proinflammatory cytokines. The overproduction of IL-17 is associated with many chronic inflammatory diseases such as, e.g. asthma [14], atopic dermatitis [15] and psoriasis [16]. On the other hand, in the case of infectious diseases, IL-17 has a positive role in the defense against pathogens, especially bacteria and fungi [12]. We did not find any study about serum levels of IL-17 in HZ in the literature, so increased IL-17 production demonstrated in our study is a new finding in HZ, whereas the activities of the aforementioned cytokines have been investigated. We found that serum IL-17 levels were significantly higher in patients with HZ than in controls.

IL-21, another cytokine evaluated in our study, is mainly produced by activated CD4 + T cells, follicular T-helper cells and natural killer T cells and targets a number of IL-21 receptor-expressing cells, primarily B, T and NK cells but also macrophages and dendritic cells. IL-21 production is increased e.g. in patients with systemic lupus erythematosus, and rheumatoid arthritis [17]. In our study, the concentration of the cytokine was higher than in the control group. As in the case of IL-17, we did not find any study about the serum levels of IL-21 in HZ in the literature.

IL-23, which was also a subject of our study, is produced by activated dendritic cells, macrophages and keratinocytes [18, 19]. It enhances the proliferation and cytotoxicity of T cells, induces the secretion of IFN-γ by T cells, affects the hematopoesis by intensifying the production of neutrophils and platelets, inhibits erythropoesis and induces the production of acute phase proteins. Previous reports have studied the expression of IL-23 serum level in collagen-induced arthritis, rheumatoid arthritis and psoriasis [19, 20]. Studies on serum levels of this cytokine in HZ patients also has not been reported.

Interestingly, in our study we observed a simultaneous increase in expression of IL-4 and IL-17, which is quite unusual, because these cytokines normally negatively regulate one another. However, a recent study by *Cooney et al*. show, that Th17 cells producing IL-17 may be resistant to suppression by IL-4, which may be associated with process of maturation or stabilization. The authors showed that after several rounds of stimulation, Th17 cells are significantly resistant to suppression by IL-4 [21]. Thus, a phenomenon which we observed in our study may be related to chronic exposure of Th17 cells to VZV antigen during periodic episodes of subclinical reactivation, which makes them resistant to inhibition by IL-4. However, further studies are needed to investigate whether this phenomenon typically occurs in HZ. Furthermore, a recently discovered a novel subset of human circulating memory CD4^+^ Th cells can produce both IL-17 and IL-4 [22].

Our result does not indicate a significant deficiency in the humoral immunity or cell-mediated immunity. The results of VZV IgG antibodies titer were significantly different in patients with HZ (x = 46,39) in comparison with control group (x = 23,86) (p<0,0001), which indicates that their production is efficient and shows a possibility of cytokines production with re-supply of the antigen. The presence of significantly higher antibodies level than in the control group demonstrates the potential efficiency of the immune system in response to a given antigen.

## Conclusions

The increased concentration of cytokines responsible for cell-mediated and humoral immunity was demonstrated. These results confirm the broad activation of the immune system involving humoral and cell-mediated immunity. It means that there is no essential impairment of cytokine production, and vaccination may be sufficient to protect against reactivation of the VZV. It is important to support recommendations for preventing HZ by vaccination of people of 50 years of age and older.

### Ethical approval

The study was approved by the Bioethical Committee of the Medical University of Białystok, Poland (reference number: R-I-002/95/2014).

## Supporting Information

S1 File Raw data. (XLSX)

S2 File Correlations. (STA)
